# Targeted RNAseq Revealed the Gene Expression Signature of Ferroptosis-Related Processes Associated with Disease Severity in Patients with Multiple Sclerosis

**DOI:** 10.3390/ijms25053016

**Published:** 2024-03-05

**Authors:** Ljiljana Stojkovic, Ivan Jovanovic, Evica Dincic, Ana Djordjevic, Jovana Kuveljic, Tamara Djuric, Aleksandra Stankovic, Slobodan Vojinovic, Maja Zivkovic

**Affiliations:** 1 Laboratory for Radiobiology and Molecular Genetics, VINČA Institute of Nuclear Sciences—National Institute of the Republic of Serbia, University of Belgrade, P.O. Box 522, 11000 Belgrade, Serbia; ljiljanas@vinca.rs (L.S.); ivanj@vinca.rs (I.J.); ana.djordjevic@vinca.rs (A.D.); jovana@vinca.rs (J.K.); tamariska@vinca.rs (T.D.); alexas@vinca.rs (A.S.); 2 Clinic for Neurology, Military Medical Academy, 11000 Belgrade, Serbia; evica.vma@gmail.com; 3 Medical Faculty, University of Defense in Belgrade, 11042 Belgrade, Serbia; 4 Department of Neurology, Medical Faculty, University of Nis, 18000 Nis, Serbia; slobodan.vojinovic@gmail.com

**Keywords:** multiple sclerosis, severity, ferroptosis, PBMCs, gene expression, targeted RNAseq

## Abstract

Detrimental molecular processes in multiple sclerosis (MS) lead to the cellular accumulation of lipid peroxidation products and iron in the CNS, which represents the main driving force for ferroptosis. Ferroptosis is an iron-dependent form of regulated cell death, with proposed roles in neurodegeneration, oligodendrocyte loss and neuroinflammation in the pathogenesis of MS. Ferroptosis-related gene expression signature and molecular markers, which could reflect MS severity and progression, are currently understudied in humans. To tackle these challenges, we have applied a curated approach to create and experimentally analyze a comprehensive panel of ferroptosis-related genes covering a wide range of biological processes associated with ferroptosis. We performed the first ferroptosis-related targeted RNAseq on PBMCs from highly distinctive MS phenotype groups: mild relapsing–remitting (RR) (n = 24) and severe secondary progressive (SP) (n = 24), along with protein detection of GPX4 and products of lipid peroxidation (MDA and 4-HNE). Out of 138 genes, 26 were differentially expressed genes (DEGs), indicating changes in both pro- and anti-ferroptotic genes, representing a molecular signature associated with MS severity. The top three DEGs, as non-core ferroptosis genes, *CDKN1A*, *MAP1B* and *EGLN2*, were replicated by qPCR to validate findings in independent patient groups (16 RR and 16 SP MS). Co-expression and interactions of DEGs were presented as additional valuable assets for deeper understanding of molecular mechanisms and key targets related to MS severity. Our study integrates a wide genetic signature and biochemical markers related to ferroptosis in easily obtainable PBMCs of MS patients with clinical data and disease severity, thus providing novel molecular markers which can complement disease-related changes in the brain and undergo further research as potential therapeutic targets.

## 1. Introduction

Multiple sclerosis (MS), a chronic inflammatory and neurodegenerative disease, is still incurable by all available treatments, which are disease-modifying and mainly target inflammation, while neurodegeneration is hard to control [1]. The pathogenesis and progression of MS are associated with the increased susceptibility of the central nervous system (CNS) to oxidative damage and mitochondrial dysfunction, due to high oxygen consumption and rich lipid content in the CNS cells. These processes lead to the accumulation of lipid peroxidation products, representing the main driving force for ferroptosis. Ferroptosis is one of the recently discovered types of regulated cell death, which is iron-dependent [2,3], and proposed to be an important player in neurodegeneration [4,5]. Moreover, among the key processes that lead to neurodegeneration in MS are the activation of microglia, chronic oxidative and axonal damage, and iron accumulation in the brain [6], all indicative of ferroptosis. Glutathione peroxidase 4 (GPX4), the enzyme that has a central role in preventing ferroptosis induction by oxidative damage, was found to be downregulated in human MS brain specimens ex vivo and in experimental autoimmune encephalitis (EAE), along with other two negative modulators of ferroptosis, cystine–glutamate antiporter (xCT) and γ-glutamylcysteine ligase (GCLC) [7]. The latter, followed by the accumulation of lipid peroxidation products and presence of abnormal neuronal mitochondria morphology, was the first evidence of ferroptotic damage in the inflammatory demyelinating lesions of MS [7]. The first experimental evidence that linked a loss of oligodendrocytes with the expression of several markers of ferroptosis was provided in a mouse model of cuprizone diet-induced demyelination [8]. Recent evidence suggests that treatment with ferroptosis inhibitors can restore the antioxidant defense and lead to a reduction in lesion size and a clinical improvement in the late stage of EAE [9]. Furthermore, it was shown that ferroptosis mediated the activation of T cells in EAE, which involved the infiltration and activation of CD4+ T cells in the CNS, while implementation of ferroptosis inhibitors led to a reduction in neuroinflammation and prevention of neuronal death in EAE mice [10]. In a novel study on MS patients, excessive lipid peroxidation products and increased iron levels, the hallmarks of ferroptosis, were found in active and chronic MS brain lesions of paraffin-embedded postmortem brain tissue blocks [11].

**Figure 1:**
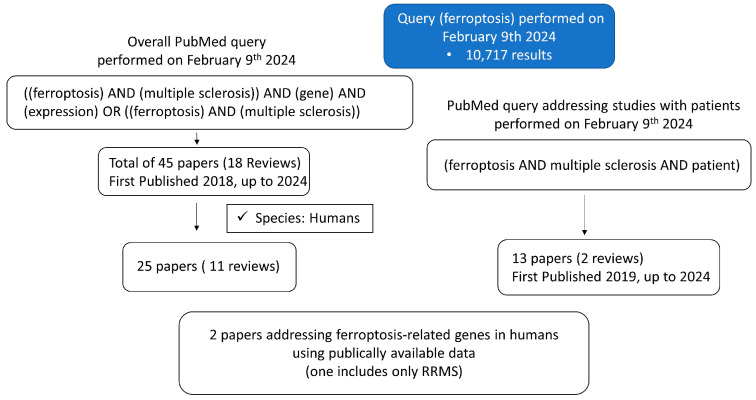
The results of PubMed search queries regarding the ferroptosis in MS.

Difficulties in comprehending the pathogenic mechanisms that cause disease progression and many uncertainties about the temporo-spatial occurrence of inflammation and degeneration in MS [12] require further research. Accordingly, ferroptosis draws attention as a biological pathway that could be modulated to achieve reductions in both inflammation and neurodegeneration in the CNS. Quite recently, ferroptosis-related pathways have been found to be upregulated in postmortem brain tissue and linked to neurodegeneration, whereby microglia were determined to be the lead player in ferroptosis-associated events in vitro [5]. Yet, as recommended by the authors of [5], the in vitro models should be complemented with the real state of disease. The experimental data about ferroptosis-related genes and their orchestration in MS patients, which possibly leads to MS progression and/or severity, are scarce. We performed PubMed searches (Figure 1) to estimate the need for further research on MS. The current literature search pointed out that although the understanding of the potential role of ferroptosis in MS is enhanced, a significant gap remains regarding studies in patients with MS.

There is still a lack of ferroptosis-specific markers and a comprehensive set of genes which would reflect ferroptosis-related molecular changes. In addition, ferroptosis-related gene expression signatures to define the set of genes whose differential expression could reflect MS severity or distinguish extreme disease phenotypes, mild relapse–remitting (RR) MS and severe secondary progressive (SP) MS, have not been identified yet. Recent efforts have been made to suggest the enrichment of biological functions associated with ferroptosis, by reanalyzing the existing transcriptome data sets in MS [13,14]. Targeted sequencing of the particular pathway and its close regulators may provide higher sensitivity for detecting both low-abundant and less variable transcripts that could be missed by a transcriptome analysis approach. This is important because relatively small changes in expression of multiple members of a particular pathway may have larger impact than a large dysregulation of a small number of genes, which is commonly detected in transcriptome analysis [15,16]. In addition, performing de novo experimental analysis in carefully defined distinctive phenotypes increases the accuracy of finding relevant and substantial differences in gene expression. As already noted [14], the main limitation of published bioinformatic studies was a lack of the clinical features of the patients, as the consequence of the employment of publicly available data sets.

Therefore, the aim of the current study was to determine and analyze the expression pattern/signature of a comprehensive set of ferroptosis-related genes in highly homogenous groups of patients with distinctive MS phenotypes. To tackle these challenges, we designed the first comprehensive panel to be investigated in MS patients, consisting of a broad range of ferroptosis-related genes (138 genes), including those with roles in lipid oxidative metabolism, antioxidant defense and iron metabolism, as well as their related main transcriptional regulators. We created a Circos plot of genes in the panel, which we selected to represent ferroptosis-related gene expression signatures with regard to related biological processes and proposed effect on ferroptosis. The gene expression was determined by targeted RNAseq in MS patients with different clinical courses of disease, long-term mild RR MS and severe SP MS, to elucidate the ferroptosis-related gene expression pattern regarding disease severity, taking into account the disease-modifying therapy. Twenty-six differentially expressed genes (DEGs) were identified between SP and RR MS patients, including changes in both pro- and anti-ferroptotic genes. Further bioinformatics methods were used for in-depth analysis and to highlight the protein–protein interaction and interplay of key DEG products, and their involvement in significantly enriched annotation terms. In addition, protein levels of GPX4, as a key antioxidant enzyme in protection against ferroptosis and lipid peroxidation products, malondialdehyde (MDA) and 4-hydroxy-2-nonenal (4-HNE) were evaluated with regard to disease severity. We verified the findings of the top three DEGs by qPCR expression in independent replication groups of RR and SP MS patients.

## 2. Results

### 2.1. Study Population

Clinical characteristics and biochemical data of the discovery and replication groups of MS patients with regard to disease course (RR and SP) are presented in Table 1. The mean age at blood sampling in the discovery group was 48.0 ± 6.2 y for RR and 49.8 ± 9.0 y for SP patients. The mean age at blood sampling in the replication group was significantly higher in SP compared to RR patients (48.5 ± 7.6 vs. 42.8 ± 8.2, respectively, *p* = 0.04). Body mass index (BMI), age at disease onset, disease duration, measured biochemical parameters in serum (iron, transferrin, ferritin) as well as GPX4 and MDA plasma levels were not significantly different between RR and SP MS patients, either in the discovery or in replication group. Concentrations of 4-HNE in plasma were significantly decreased in SP MS patients in the discovery group, while there was no significant difference between the two disease phenotypes in the replication group. The Expanded Disability Status Scale (EDDS), Multiple Sclerosis Severity Score (MSSS), total number of relapses and number of relapses during the last two years were significantly different between RR and SP in both the discovery and replication groups, with regard to the cutoff values set as inclusion criteria. In the discovery group, there were significantly more men in the RR patients compared to SP patients, while in the replication group, RR and SP MS patients were sex-matched (Table 1). The EDSS and MSSS scores did not differ significantly between males and females within the RR and SP patients, both in discovery and replication groups.

### 2.2. Differentially Expressed Ferroptosis-Related Genes in PBMCs between SP and RR MS Patients

**Figure 2:**
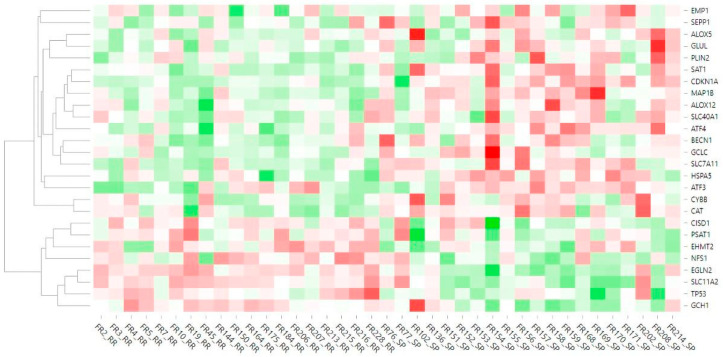
Heatmap of gene expression data. The heatmap represents relative expression of ferroptosis-related DEGs across the samples and their clustering. Red color indicates overexpression while green color shows downregulation. White color shows the middle expression of the gene across the samples.

Analysis of the data obtained from the targeted sequencing of 19 RR and 20 SP MS patients, retained after outlier removal, led to the identification of 26 DEGs according to nominal *p* value (Table 2). In the set of identified DEGs, 18 genes were upregulated while 8 genes were downregulated in SP patients compared to RR patients. Graphical representation of the DEGs’ relative expression across the samples is demonstrated on the heatmap (Figure 2). Introduction of disease-modifying therapy status as a secondary factor in the analysis model did not result in changes in key differentially expressed genes (Supplemental Table S2).

**Figure 3:**
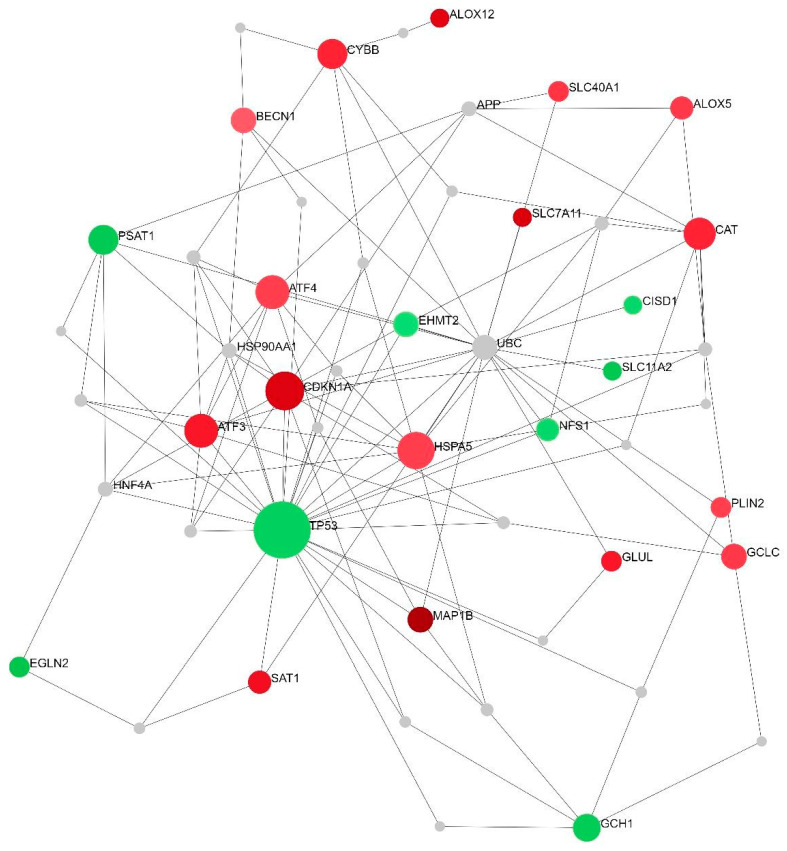
A minimum-order PPI network of all ferroptosis-related DEGs between SP and RR MS patients. A minimum-order network was woven by taking into account the IMEx Interactome data as a background. Green color represents downregulated genes while red color represents upregulated genes in SP compared to RR MS patients. The size of the nodes depicts the level of interactions, thus emphasizing major network hubs.

**Figure 4:**
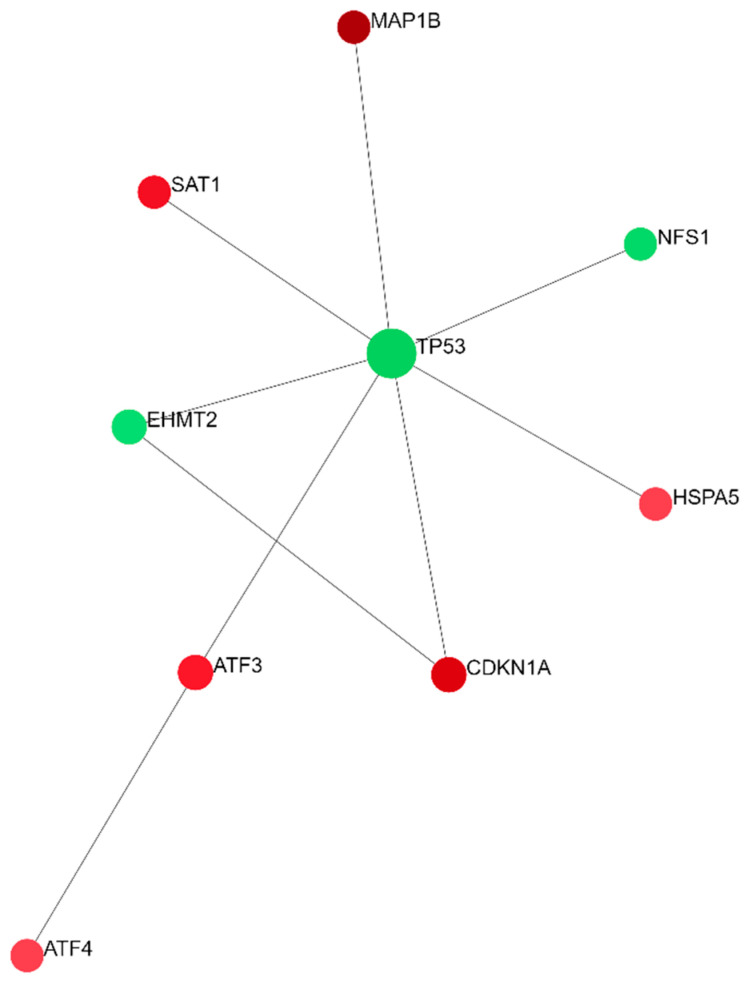
A zero-order PPI network of all ferroptosis-related DEGs between SP and RR MS patients. A zero-order network was woven using the Network Analyst tool by taking into account the IMEx Interactome data as a background. Green color represents downregulated genes while red color represents upregulated genes in SP compared to RR MS patients.

To identify the relationships between all DEGs implicated in ferroptosis-related processes, the NetworkAnalyst 3.0 online platform was applied for generating networks of protein interactions. Of the entire set of DEGs (n = 26), 24 were successfully integrated as seeds of a minimum network containing 51 nodes and 117 interactions (Figure 3). There were two seeds among three nodes with the highest centrality degrees: TP53 with the maximum number of interactions, 28, and CDKN1A with 11 interactions. The enrichment analysis performed on the minimum network confirmed the strong enrichment of the ferroptosis pathway (Table 3). A zero-order network has retained only the direct interactions in the Interactome database (Figure 4). Of the top three DEGs, two were also identified in the zero-order network (CDKN1A and MAP1B), forming an independent axis with a TP53 acting as a hub molecule (Figure 4).

**Figure 5:**
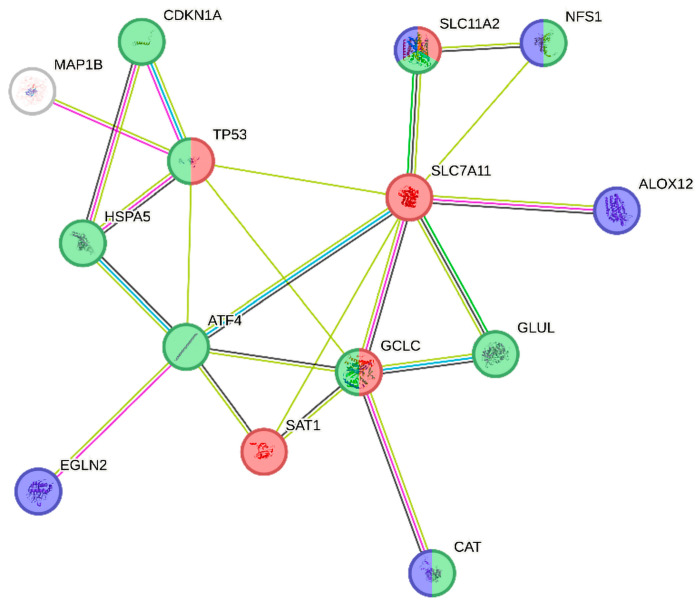
STRING PPI network of ferroptosis-related DEGs between SP and RR MS patients. The STRING v12 database was employed on a set of DEGs passing correction for multiple testing. Colors depict members of the top-enriched annotation terms in the network, computed by the STRING database. Red: ferroptosis (KEGG), Green: catalytic complex (Gene Ontology: Cellular Component), Blue: iron (Annotated key words UniProt). Edges represent protein–protein associations representing joint contributions to a shared function as described on https://string-db.org/.

For further visual exploration, the STRING v12 database was employed on a set of DEGs passing correction for multiple testing (Figure 5). The computed PPI enrichment *p* value of 1.81 × 10^−7^ indicated that network proteins have significantly more interactions among themselves than what would be expected for a random set of proteins of the same size and degree distribution drawn from the genome. Further functional annotation of the network has indicated significant enrichment of the ferroptosis pathway (KEGG), catalytic complex (Gene Ontology: Cellular Component—GO:CC) and iron (UniProt Annotated Keywords), which were employed for differential visualization of the network nodes (Figure 5). All of the annotation terms were top-enriched for the corresponding databases (Supplemental Table S3). It is worth noting that one of the top three DEGs, MAP1B, was not only unassigned to the top-enriched terms, but it was not even a member of any of the enriched terms presented in Supplemental Table S3.

**Figure 6:**
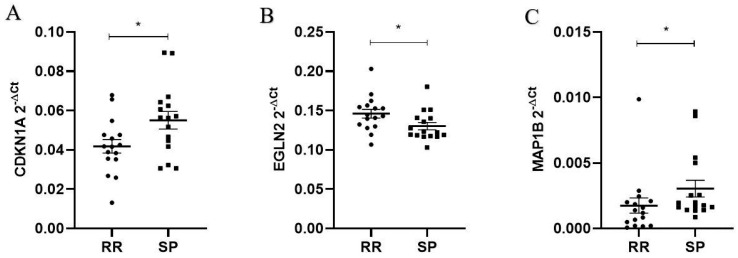
Relative expression of *CDKN1A, EGLN2* and *MAP1B* mRNAs in the replication group of RR and SP MS patients’ PBMCs. Relative mRNA expression is presented as 2^−∆Ct^ values for each sample. The cDNAs from human PBMCs (N = 32) were used to quantify gene expression. The delta Ct value was calculated from the difference between the Ct value of the gene of interest and that of the reference gene, *PPIA*. Data are presented as 2^−∆Ct^ as mean for both groups (RR-circles and SP-squares) ± SEM. The difference of mRNA relative expression between groups was calculated using a Mann–Whitney *U* test. (**A**) Significant upregulation of *CDKN1A* mRNA was detected in PBMCs from SP patients (N = 16) compared to RR patients (N = 16) (0.05507 ± 0.00565 vs. 0.04182 ± 0.00565, *p* = 0.04). (**B**) Significant downregulation of *EGLN2* mRNA was detected in PBMCs from SP patients (N = 16) compared to RR patients (N = 16) (0.13020 ± 0.00732 vs. 0.14600 ± 0.00732, *p* = 0.01). (**C**) Significant upregulation of *MAP1B* mRNA was detected in PBMCs from SP patients (N = 16) compared to RR patients (N = 16) (0.00305 ± 0.00087 vs. 0.00175 ± 0.0008, *p* = 0.04); * *p* < 0.05.

The relative expression of the top three differentially expressed genes (*CDKN1A*, *MAP1B* and *EGLN2*), identified using RNAseq analysis, has confirmed the dysregulation of their mRNA levels in the replication group of RR and SP MS patients’ PBMCs by qPCR (Figure 6). We analyzed age as a possible confounder and found no significant effect on gene expression in the replication group.

## 3. Discussion

In this study, we performed targeted mRNA sequencing of the comprehensive set of ferroptosis-related genes in two extreme MS phenotypes: mild RR and severe SP MS. An exponential rise in the number of studies related to ferroptosis has occurred in the past two years, but still a limited number of papers have presented new original experimental data, particularly in patients and with regard to neuroinflammation and neurodegeneration. Most of the studies related to MS pathology were performed in the EAE model, animal model of MS and several types of cell models. In humans, bioinformatic analyses of ferroptosis genes in the pre-existing transcriptome datasets predominate [13,14]. There is a lack of studies conducted in MS patients encompassing clinical features and disease severity. Ferroptosis emerged as a relevant form of regulated cell death because it is iron-dependent and could drive neuro-axonal loss, which leads to still untreatable neurodegeneration in MS. Recently, in the context of ferroptosis, accumulating levels of Fe^2+^ were detected in active MS lesions and in the rims of chronic lesions, while the Fe^2+^/Fe^3+^ ratio was found to be increased in the CSF of MS patients [11]. The total iron content and MDA in CSF were not significantly different in MS compared to non-MS controls, which is similar to our results obtained between RR MS and SP MS patients, in blood. The immunopathophysiological view of MS has recently been updated, pointing out that the interaction of peripheral and CNS cells occurs in both ways, enabling the influence of CNS-produced molecular cargo to affect peripheral immunity [17]. In peripheral immune cells of MS patients, many endogenous factors have pleiotropic and redundant functions and act in complex networks, which could result in subtle molecular changes that are hard to detect [18]. Hence, the plausible approach to detect the ferroptosis-related gene expression differences in PBMCs of MS patients with regard to disease severity was to apply targeted RNAseq, which offers multiple advantages, enabling higher transcript sensitivity and higher throughput of samples [15,16,18] in comparison to transcriptome-wide study. Out of 138 selected ferroptosis related genes, including their main transcriptional regulators, 26 DEGs were identified between severe SP and mild RR MS patients. As expected, the identified DEGs were those that code for key components of the main ferroptosis-related processes, including lipid peroxidation: *ALOX5* and *ALOX12*; cellular iron utilization and import/export: *NOX2/CYBB*, *CAT*, *SLC11A2* and *SLC40A1*; and xC^−^/GPX4-dependent antioxidant defense system: *SLC7A11* and *GCLC*. The pathway enrichment analysis performed on an interaction network composed of the DEGs confirmed the strongest enrichment of the ferroptosis pathway. Intriguingly, the most prominent difference between RR MS and SP MS was related to genes known to regulate cell cycle, cell proliferation, apoptosis and resilience to ferroptosis in cells under stress: *CDKN1A*, *MAP1B*, *SAT1*, *EGLN2* and *TP53*. To increase the validity of the findings, we performed replication of the top three DEGS (*CDKN1A*, *EGLN2*, *MAP1B*) in an independent cohort of RR MS and SP MS patients.

The main obstacle in the research and treatment of MS is finding biomarkers and therapeutic targets in the periphery that accurately reflect and/or influence the CNS processes. Translation of results from in vitro and in vivo models to a clinical setting is the most challenging task. Among the DEGs identified in our study, several are already proposed as potential therapeutic targets, mainly in cancer research. In contrast to cancer, where the main goal is to selectively target cancer cells by inducing ferroptosis, in neurodegenerative diseases, the attenuation of ferroptosis is required to prevent or delay neuronal cell death and disease progression. Lipid peroxidation and dysregulation of iron homeostasis are key triggers of ferroptosis. We identified increased expression of the important players in lipid metabolism, *ALOX5*, *ALOX12* and *SAT1*, in the PBMCs of patients with SP MS in comparison to the mild RR phenotype. The role of the *ALOX5* gene in MS, found to be upregulated in both MS and EAE white matter inflammatory lesions [19], was recognized even before the term ferroptosis was defined. ALOX5 is a key enzyme in the biosynthesis of proinflammatory leukotrienes from arachidonic acid, which represent major players in neuroinflammation [20]. ALOX12 metabolizes arachidonic acid and other polyunsaturated fatty acids to corresponding lipid hydroxides that participate in the pathogenesis of neurodegenerative diseases [21]. Earlier studies revealed that ALOX12 played a key role in neuronal cell death [22] and death of oligodendrocytes caused by glutathione depletion-induced toxicity and accumulation of ROS [23]. Recent findings have shown that the inhibition of ALOX5 attenuated the accumulation of lipid peroxides and neuronal damage and prevented ferroptosis in a model of Parkinson’s disease [24]. Another pro-oxidant gene that we found to be upregulated in SP MS, *NOX2/CYBB*, is proposed to be an important contributor to oxidative stress and subsequent neurotoxicity in the CNS [25]. Besides its contribution to microglia-related EAE disease severity and neuronal damage [26], the sensitization to ferroptosis by activation of NOX2 was suggested in cancers [27]. With regard to iron regulation and utilization, a recent study has presented the dysregulated expression of genes involved in these processes, following increased lipid peroxidation in a chronic EAE, but not a relapsing–remitting EAE model [9]. The complexity of disease, including relapse, remission and progression phases, was reflected by gene expression changes through stages of EAE, showing the peak of *SLC11A2/DMT1* expression at the peak of chronic EAE, decreasing to pre-onset levels in the progressive stage. Another recent study has shown that transcriptional changes related to ferroptosis were more prominent during acute EAE and relapse, while demyelination progressively increased over time [11]. Expression of *GPX4*, often assigned as the hallmark defense enzyme in ferroptosis that is required for glutathione-mediated antioxidant defense, remained unchanged between RR and chronic EAE [9]. The *GPX4* gene expression and plasma protein levels were not different between MS phenotypes in our study. Although 4-HNE, as a lipid peroxidation product, was not increased in the severe MS phenotype in our study, values in both groups were higher in comparison to previously published values in healthy individuals, both measured by ELISA [28]. We also found differences in iron-related genes, including lower *SLC11A2* and higher *SLC40A1* mRNA levels in PBMCs of SP MS patients, compared to RR MS.

The main cellular functional component that protects from ferroptosis is the cysteine−glutamate antiporter (Xc−)−GPX4-dependent antioxidant defense system [2]. In line with unchanged *GPX4*, expression of other genes involved in this ferroptosis-protective axis was not diminished in SP MS. Moreover, we detected significantly higher expression levels of *SLC7A11 (xCT)* and *GCLC* genes in PBMCs of SP, compared to RR, MS patients. Previous findings about the role of SLC7A11 in neuroimmune processes are inconsistent. Several studies demonstrated that upregulation or activation of xCT enhanced the autoimmune inflammatory demyelination [29,30,31]. In addition, *xCT* mRNA and protein expression levels were upregulated in EAE and MS, both in the CNS and the monocyte–macrophage–microglia lineage [30], while inhibition of xCT attenuated chronic and RR EAE in terms of abrogating the clinical disease and attenuating T cell infiltration, inflammation and myelin loss in the CNS [31]. On the other hand, an increase in *SLC7A11* expression in the CNS was associated with decreased levels of tissue iron and the lipid peroxidation product malondialdehyde, increased glutathione, as well as inhibited neuronal and microglial ferroptosis [32,33]. Our results showing the upregulated expression of *SLC7A11* and *GCLC* genes could assume their compensatory activation toward the support of glutathione-related antioxidant effects on PBMCs in SP MS patients, which is in line with an absence of decrease in *GPX4* levels. In support of our assumption, induction of oxidative stress, a relevant contributor to MS progression, caused an increase in *GCLC* mRNA levels in the spinal cord [34]. An important role of GCLC and glutathione was evidenced in neuroprotection [35]. Given that xCT and GPX4 are currently the main therapeutic targets in controlling ferroptosis, further research into neuroinflammatory and neurodegenerative diseases is warranted. *CAT* gene expression was also significantly increased in PBMCs of SP MS patients. This could be explained as a compensatory response to counteract oxidative stress-caused elevated levels of ROS [36], which might represent a consequence of upregulation of the above-mentioned pro-oxidant genes. Accordingly, CAT and other antioxidant enzymes were markedly upregulated in the active demyelinating lesions from patients with progressive MS, in comparison to normal-appearing white matter and white matter tissue from control brains [37]. Recently, a GPX4/glutathione system-independent regulation of ferroptosis resistance has been discovered, describing the GCH1 as the central, tetrahydorbiopterin (BH4) rate-limiting enzyme [38]. The *GCH1* gene was described as the key protective gene that suppressed ferroptosis, whose overexpression showed a potent antiferroptotic effect [38]. In our SP MS patients, it was significantly downregulated in comparison to RR MS. One of the proposed consecutive effects of MS is the decrease in BH4, which can disrupt antioxidative defense and promote disruption of the blood–brain barrier [39].

Novel findings indicate that even if inflammatory activity decreases over the progressive course of MS, the chronic active lesions and low-grade inflammation persist in SP MS, leading to worsening of disability [40]. One of the strengths of our study is providing novel data in the SP MS phenotype, addressing the disease severity in relation to ferroptosis, independently of the relapse phase, which was shown to affect ferroptosis-related transcription profiles. Not many studies have investigated gene or protein expression changes related to ferroptosis in peripheral cells in humans. Hence, there are still many uncertainties about how the processes of the cascade of ferroptosis-associated events work and how they affect different tissues and cell types. Several studies have investigated ferroptosis mechanisms in oligodendrocytes, as their loss is one of the hallmarks of progressive MS. Oligodendrocyte loss and demyelination were associated with ferroptosis [8]. Yet, in a state of metabolic stress, ferrostatin-1, a ferroptosis inhibitor, did not prevent oligodendrocyte death [41]. However, resistance of oligodendrocytes to oxidative stress, in comparison to HeLa cells, was characterized by the upregulation of *CDKN1A*, a gene responsible for reduction in sensitivity to ferroptosis, and *SAT1*, a key metabolic regulator in the catabolism of polyamines previously related to ferroptosis [42]. The important novelty in our study was the differential expression of the top three genes in the discovery group. We found that *CDKN1A/p21* and *MAP1B* were upregulated, while *EGLN2* was downregulated, in SP compared to RR MS patients. Considering their pleiotropic roles and the fact that they are not the core but the ferroptosis-related genes, we performed the replication in an independent group of patients. Differences in gene expression, obtained in the discovery group, were confirmed in the replication RR MS and SP MS cohorts. Herein, the observed change in the expression of *CDKN1A/p21*, coding for a member of a family of cyclin-dependent kinases inhibitors and acting as a cell-cycle inhibitor [43], could result in altered CD4+ T cell activation and proliferation [44]. Additionally, an increase in *SLC7A11* expression in our SP MS patient group could be related to p21, since p21-mediated activation of the *SLC7A11* promoter was evidenced, providing redox control and regulation of neuronal cell death [45]. EGLN2, a prolyl hydroxylase domain protein, representing an oxygen sensor and a critical regulator of the response to hypoxia [46], could disturb redox homeostasis through reducing oxidative pentose phosphate pathway flux and increasing the oxidized/reduced glutathione ratio [47]. It was suggested that the EGLN family could exert both pro-oncogenic and antitumorous effects [48], and that EGLN2 had a role in affecting mitochondrial homeostasis [49]. Along with these genes, *SAT1* was differentially expressed showing upregulation in SP MS. Activation of *SAT1* expression can induce lipid peroxidation and sensitize cells to undergo ferroptosis upon reactive oxygen species-induced stress [42]. Both *CDKN1A/p21* and *SAT1* are *TP53* target genes [50]. Protein–protein interaction network analysis of all ferroptosis-related DEGs between SP and RR in the current study showed that TP53 had the highest number of interacting partners, including the aforementioned top DEGs, *CDKN1A/p21*, *MAP1B* and *SAT1*. The tumor suppressor protein p53 influences anti-oxidant response [51]. It was shown that a delay of ferroptosis in cancer cells required the *CDKN1A/p21*, and that the p53-p21 axis was associated with a slower depletion of intracellular glutathione and a reduced accumulation of lipid ROS [52]. The exact mechanism of how the p53-p21 axis relates to ferroptosis is not known yet, but the experiments suggest that it is independent from nuclear factor erythroid 2-related factor 2 (NRF2) activity. This is important because NRF2 is a proposed molecular therapeutic target, which can promote or inhibit ferroptosis through modulation of intracellular signaling. In our study, *NRF2* was not significantly different among groups, and *TP53* was downregulated in SP MS. Similar to our results, an inverse change in p53 and p21 levels in the PBMCs of patients with Alzheimer’s disease was revealed [53], and the suppression of the *p53* gene was accompanied by the induction of *MAP1B* (microtubule-associated protein 1B) during neuronal differentiation in vitro [54]. The same relation between *TP53* and *MAP1B* was detected in PBMCs of SP MS in the current study. Moreover, it was shown that MAP1B negatively regulated p53-dependent transcriptional activity, which was driven by the *p21* promoter [55]. Although *MAP1B* is predominantly expressed during the early stages of development of the nervous system, with a role in axonogenesis [56], it is also important for axonal plasticity and regeneration in the adult nervous system [57,58]. The functional importance of MAP1B in CNS myelination was noted by observing the defects in the myelin development in *MAP1B* knock-out mice [59]. However, in a giant axonal neuropathy model, overexpression of MAP1B in cortical neurons led to development of cell death characteristics, suggesting its link with neurodegeneration [60] and promotion of cell death in diseases such as Alzheimer’s [61]. Accordingly, the effects of MAP1B on CNS structural and functional characteristics may be beneficial or adverse, depending on condition and cellular context. We discovered and replicated *MAP1B* overexpression in PBMCs of SP MS in comparison to RR MS, suggesting its role in neuroimmune mechanisms, which can be detected in the periphery and has not been established so far.

The strength of the current study is that patients who were included were carefully selected to fulfil the criteria of long-term mild RR MS, to minimize the possibility of disease progression and without recent relapses that could influence acute changes in gene expression profiles, as shown in animal models. In addition, disease-modifying therapy was included as a second factor in differential expression analysis and was not found to interfere with the expression signature detected among phenotypes. Probably the main limitation of the current study is the lack of correlation of genetic data with brain imaging. It would provide deeper insight into linking molecular changes in the periphery with disease activity in the brain and would strengthen the interpretation of observed gene expression changes and improve further selection of target molecules with regard to disease modification. However, such results are still lacking. The sample size of the discovery group, which can always be considered a limitation, particularly in heterogenous diseases such as MS, can to a certain extent be overcome by the selection of homogenous groups and a replication of the most significant genes in an independent cohort. The current study uses the better off previously performed transcriptome comparisons of MS patients with different courses of disease, which were mostly performed in the period when ferroptosis was not assigned as one of the pathways in KEGG. Recently reanalyzed transcriptome data suggested the role of ferroptosis in MS, but in cohorts not controlled for heterogeneity and clinical data. In addition, application of RNAseq and targeted analysis of a selected set of genes in comparison to microarray transcriptome screening, exceeds the need for validation of the results by qPCR, as the methodology is more sensitive to quantifying both low-abundant and less variable transcripts that could be missed by a transcriptome analysis approach. However, it does not exclude the need for further functional mechanism investigations.

To our knowledge, no studies have investigated the relationship between ferroptosis-related genes and SP MS, along with lipid peroxidation products and a hallmark protein of ferroptosis, GPX4, in the peripheral blood. Progression of MS, which occurs despite disease-modifying therapies is a great current burden for both patients and researchers. CNS cell models and postmortem brain analysis have shown signs of ferroptosis but studies in peripheral cells and patients are still scarce. Our study integrates wide genetic signature and biochemical markers related to ferroptosis in the easily obtainable PBMCs of MS patients with clinical data and disease severity, thus providing novel molecular markers, which can complement disease-related changes in the brain and undergo further research as potential therapeutic targets. Since ferroptosis targeting showed a substantial capacity during the last two years in several diseases, it is of great importance to define the state of the ferroptosis-related molecules in different phases and courses of MS. In secondary progressive patients, we identified differential expression of genes related to lipid peroxidation, antioxidative defense and those that regulate cell cycle, cell proliferation, apoptosis and resilience to ferroptosis, suggesting the mirroring of the CNS recognized processes in the periphery. The complexity of the ferroptosis cascade requires further understanding of in-depth mechanisms and validation in clinical settings. We presented co-expression and interactions of DEGs, which are additional valuable assets for deeper understanding and research of ferroptosis-related genetic machinery in MS. It is noteworthy that the currently presented panel for targeted mRNA sequencing can be advantageous for future studies where the primary interest lies in the ferroptosis pathway.

## 4. Methods and Materials

### 4.1. Subjects

The discovery study group included 48 unrelated patients from Serbia, 24 with relapsing–remitting and 24 with secondary progressive MS, recruited from the Clinic for Neurology of Military Medical Academy (MMA), Belgrade, Serbia, and the Neurology Clinic of University Clinical Center Nis, Nis, Serbia, during the period from 2022 to 2023. Recruitment of patients was carefully planned to enable selection of two extreme and homogenous MS phenotype groups, a group with mild disease and minimal neurological disability, RR, and a group with progressive disease with severe neurological disability, SP. Patients were recruited during their regular visits to clinics where they were treated and regularly followed up by the same neurologist. Diagnosis of clinically definite MS was performed according to the revised McDonald criteria [62,63]. In RR patients, a pronounced inflammatory component of the disease was presumed [64,65]. The course of disease was defined according to a clinical method [66,67] to precisely characterize the SP MS group, with a presumed pronounced neurodegenerative component of the disease [64,65]. None of the patients in the study group relapsed during a period of at least 30 days prior to the study enrollment, and thus none of them had been recently treated with corticosteroids. For each patient, a detailed questionnaire was filled out based on previous clinical records and an interview was performed by a neurologist to provide accurate data on demographic (age, sex, BMI) and clinical parameters: age at onset, disease duration, total number of registered relapses, number of relapses during two years before inclusion in the study, and previous and ongoing therapy. Clinical parameters were determined at the time of peripheral blood sample collection. Neurological impairment was scored by the same trained and experienced neurologist, using the EDSS [68]. The absence of relapses within the previous month minimized the possibility of EDSS improvement in the remission phase following the relapse. To improve the estimation of disease severity, we applied the calculation of the MSSS, which corrects the EDSS for disease duration [69]. The cutoff values for inclusion were EDSS < 2 for RR MS patients and EDSS > 6 for SP MS, and a disease duration of minimum ten years in all patients. Although we designed the study to include only mild RR patients and thus minimize the possibility that they progressed to SP, we included only those that had long-term, at 10 years, mild disease and were regularly followed. Mild RR patients were selected among those in whom treatment started soon after the diagnosis (more than ten years ago), and the limited treatment options at the time made sex matching in the RR discovery group limited. The replication group (16 RR and 16 SP MS) for gene expression analysis of the top three differentially expressed genes (DEGs) identified in the discovery group was recruited in a way to maximally resemble the discovery group. All diagnostic criteria applied for this group were identical to the discovery group and the same questionnaires were filled in. It was challenging to recruit such an extreme MS phenotype of both mild and severe patients, like in the discovery group. The vast majority of MS patients with disease duration of 10 years have an EDSS score of at least 3, as shown in a study that included >25,000 MS patients [70]. Although the patients in the replication group had five years shorter disease duration on average, the mean disease duration was above 10 years. For the progressive SP group, we lowered the inclusion criteria for EDSS to ≥5, since severe patients are harder to reach due to their high disability and are less willing to accept participation in research studies.

The MMA Ethics Committee approved the study (Decision No 6/2020). Each participant gave their written informed consent for participation in the study.

In the discovery cohort, out of 24 selected RR patients, 21 were under low-risk, moderate-efficacy therapy (18 treated with interferon beta, 2 with glatiramer acetate and 1 with teriflunomide). All of them were receiving long-term therapy. Among 24 SP patients, 12 had received immunomodulatory treatment for a period of at least 12 months in the past, following the more efficacious therapies after disease breakthroughs or stopping the treatment on several accounts. At the time of enrolment in the study, they were under disease-modifying treatments. The other half of SP patients were treatment-naïve from the beginning of the disease. In all of the SP patients, the disease had progressed despite the therapy. In all patients, blood was drawn before receiving the next dose of therapeutic.

### 4.2. Curated Selection of the Ferroptosis-Related Genes

**Figure 7:**
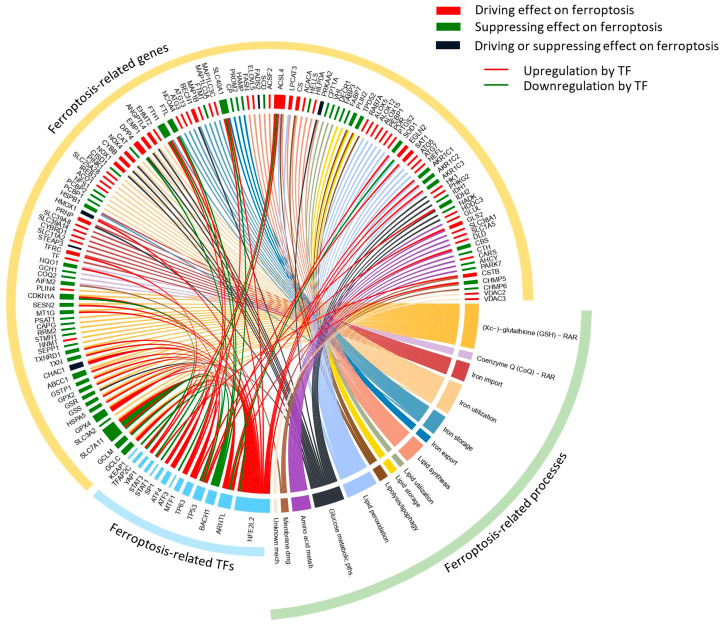
Circos plot of the ferroptosis-related genes selected for RNAseq panel. The Circos plot represents selected ferroptosis-related genes (yellow outer ribbon) with regard to the ferroptosis-related processes (green outer ribbon) and ferroptosis-related transcription factors (blue outer ribbon), which were included in the RNAseq panel. Within the inner circle, gene-associated rectangles are colored according to the proposed effect on ferroptosis: driving effect (red), suppressing effect (green), driving or suppressing (black). Rectangles associated with transcription factors are represented with a blue color. Rectangles associated with ferroptosis-related processes are represented with a unique color clearly distinguishing all of the presented processes. The size of rectangles in the inner circle depicts the number of associations within the Circos plot while the color of the line presents the link of the selected genes with the particular ferroptosis-related processes. Red and green lines depict upregulation and downregulation of selected genes by transcription factors, respectively. The plot was generated according to the data presented in the Supplemental Table S1.

Selection of the genes for the ferroptosis-related targeted mRNA sequencing panel initially considered genes from the ferroptosis pathway map (hsa04216) of the Kyoto Encyclopedia of Genes and Genomes (KEGG) Pathway Database (https://www.genome.jp/pathway/hsa04216) [71,72]. Further, we performed extensive literature and database research (FerrDb, http://www.zhounan.org/ferrdb/current/) (accessed on 29 December 2023) [73] to complementarily examine the wider genetic background of ferroptosis-related processes and expand the range of selected genes relative to the core KEGG ferroptosis pathway (Figure 7). The recently developed FerrDb offers a substantial foundation for searches of ferroptosis-related genes. However, the extremely dynamic expansion of the data in ferroptosis research requires additional efforts to curate data and more importantly to classify the role of the proposed relevant genes. Consequently, FerrDb is growing, although many genes are still unclassified and experimentally detected in a limited number of studies. To supplement current knowledge, we manually selected and carefully curated panel genes so as to directly or indirectly participate in the relevant ferroptosis-related processes, encompassing antioxidant defense (Xc−-GSH-GPX4 and CoQ defense systems), metabolic processes and pathways (iron, lipid, glucose and amino acid metabolism) and cell membrane damage/repair mechanism. Beyond those previously mentioned, we included additional genes based on the most recent experimental evidence relevant to ferroptosis, such as euchromatic histone lysine methyltransferase 2 (*EHMT*2, also known as *G9a*) [74] and Kelch-like ECH-associated protein 1 (*KEAP1*), as the main transcriptional regulator of NFE2-like BZIP transcription factor 2 (*NFE2L2*/*NRF2*). All of the selected genes were further inspected for known general gene expression levels in the whole blood, represented in the Genotype-Tissue Expression (GTEx) Portal (https://www.gtexportal.org/home/gene) [75] and GeneCards expression section [76] (https://www.genecards.org/) (accessed on 29 December 2023). Finally, the created custom panel for targeted mRNA sequencing involved 138 genes. The genes were classified according to their roles in ferroptosis-related processes together with their proposed direct/indirect effect on ferroptosis and are described in detail in Supplemental Table S1. Among the 138 selected panel genes, 14 encoded transcription regulators were associated with ferroptosis. They were sorted into those that affected the transcription of either multiple genes directly/indirectly involved in the stated ferroptosis-related process(es) or a single gene directly involved in a key ferroptosis-related process [77,78,79]. The complete panel of selected genes with regard to the ferroptosis-related processes in which they are involved and their proposed effects on ferroptosis are presented in a Circos plot generated using the *circlize* package for circular visualization in R [80] (Figure 7).

### 4.3. Design of the AmpliSeq Illumina Custom RNA Panel for the Profiling of Ferroptosis-Related Genes Using Targeted RNAseq

To design a custom RNAseq panel consisting of a pool of amplicons covering the previously prioritized target genes, Illumina Design Studio was used. To design an optimized panel, which allows efficient and balanced sequencing of all requested targets, an insight into the previously documented expression of selected target genes in whole blood and white blood cells [75,76] (https://www.gtexportal.org/home/gene; https://www.genecards.org) (accessed on 29 December 2023) was performed. Additionally, Illumina Design Studio offers a functionality that controls for highly expressed genes in the panel, and suggests additional consideration before making a decision to enroll these genes in the panel. After analysis of documented mRNA expression in normal human tissues we decided to generate one pool of primers that contained 127 genes (large sub-panel) and the second panel consisted of 11 genes that were marked as “highly expressed” (small sub-panel). Two separate sequencing libraries were generated with accompanying manifest files for successful mapping of AmpliSeq-generated reads.

### 4.4. Isolation of PBMCs and Extraction of the Total RNA

The PBMCs were isolated from the peripheral blood samples using lymphocyte separation medium (PAA, GE Healthcare, Chicago, IL, USA), and the total RNA was extracted from PBMCs by using TRI Reagent (Ambion, Life Technologies, Austin, TX, USA). Extracted total RNA samples were dissolved in the nuclease-free water (Ambion) and stored at −80 °C. Total RNA concentration and purity were determined with a NanoDrop ND-1000 spectrophotometer (Thermo Scientific, Waltham, MA, USA).

### 4.5. Targeted RNAseq Library Synthesis

Synthesis of sequencing libraries was performed according to the AmpliSeq™ Library PLUS for Illumina^®^ using PBMC RNA samples of 48 MS patients (24 RR samples and 24 SP samples). In brief, 50 ng of total RNA underwent reverse transcription using AmpliSeq cDNA synthesis for Illumina kit. Using previously designed custom panels, targeting regions were amplified and partially digested. Before the ligation of indexes onto individual libraries, pooling strategies were made to achieve optimum color balance for AmpliSeq™ CD Indexes Set A for Illumina^®^ by following the Illumina Index Adapters Pooling Guide. After the indexing procedure, the first step of library cleanup was performed using AMPure XP Beads reagent (Beckman Coulter, Brea, CA, USA), followed by library amplification and the second cleanup step. Resuspended libraries were quantified on Qubit 3.0 Fluorometer using a Qubit DNA HS Assay Kit. The molarity of the libraries was calculated according to the AmpliSeq™ Library PLUS for Illumina^®^ protocol instructions by using 350 bp as the average library size. Libraries were diluted and pooled at final loading concentration of 50 pM. Pooled libraries contained 8 samples per sequencing run (4 RR samples and 4 SP samples) for the “large sub-panel”, while 24 libraries generated with the “small sub-panel” were pooled per sequencing run (12 RR samples and 12 SP samples).

### 4.6. Targeted RNA Sequencing and Data Analysis

Pooled libraries were sequenced on an iSeq™ 100 System with a run configuration of 2 × 151 bp. All sequencing runs were performed with the same settings defined in Local Run Manager software version 2.4.0.2466. The settings were defined as follows: Module: RNA Amplicon—1.0.2; Workflow: RNA Amplicon; Library Prep Kit: AmpliSeq Library PLUS for Illumina; Chemistry: Amplicon. The appropriate manifest files were uploaded in order for secondary analysis to be performed automatically after the sequencing run was completed. During the sequencing run, primary analysis parameters were monitored by the in-house responsible staff using Illumina Sequencing Analysis (SAV) viewer. After the run was completed and demultiplexing was performed, additional parameters were included in quality check: indexing QC (to control for successful library normalization and pooling), phasing and prephasing (the estimated percentage of molecules in a cluster for which sequencing falls behind (phasing) or jumps ahead (prephasing) of the current cycle within a read) and the total number of reads passing filter. FASTQ generation was performed on Illumina BaseSpace cloud while mapping of reads onto sequences contained in manifest files, metadata acquirement and read cleanup and alignment were performed on-instrument using previously uploaded manifest files.

Secondary analysis was automatically performed on-instrument according to the RNA Amplicon module (Local Run Manager, RNA Amplicon Analysis Module, Illumina Document # 1000000048048 v03). Quality checks included the monitoring of % of reads mapping onto target regions and distribution of reads on target regions using aggregated summary of the secondary analysis. After all quality checks were performed, raw read counts were exported from the instrument for further, tertiary analysis.

Tertiary analysis for identification of differentially expressed target genes (DEGs) was performed using Galaxy server https://usegalaxy.eu (accessed on 29 December 2023) [81] and DeSeq2 workflow [82] using previously obtained raw read counts. The computed size factors and generated Principal Component Analysis (PCA) plots were used in conjunction for the identification and exclusion of outlier samples, coupled with the DeSeq2 outlier replacement (when there are more than 6 replicates for a given sample, the DESeq2 will automatically replace counts with large Cook’s distance with the trimmed mean over all samples, scaled up by the size factor or normalization factor for that sample). The described workflows were performed for both “small sub-panel” and “large sub-panel” analysis of DEGs. In addition, DeSeq2 workflow was performed by including therapy status as a secondary factor to correct for potential influence on DEGs between primary factors (SP vs. RR MS).

The Rlog-transformed expression data of the differentially expressed genes were used for generating a heatmap using the expression module of the Heatmapper tool [83]. Pearson correlation was used as a distance metric, and the average linkage method was subsequently employed for hierarchical clustering of the genes.

### 4.7. Bioinformatic Analysis

To investigate relationships between the all of the identified DEGs, a protein–protein interaction (PPI) network was constructed using the NetworkAnalyst 3.0 [84] online platform https://www.networkanalyst.ca (accessed on 29 December 2023) [85] based on the IMEx Interactome database of literature-curated comprehensive data from InnateDB [86]. Each network node was characterized by its number of connections to other nodes, defined as the degree of a node. The network analysis identified nodes with a high degree, which represented hubs within the created network. The minimum and zero-order networks were created to keep only those nodes that were necessary to connect the seed nodes (uploaded set of DEGs), or only the seed nodes, respectively [84]. Gene set overrepresentation analysis, and enrichment analysis supported by NetworkAnalyst, were applied to identify pathways from the KEGG database, which had a significant overlap with network DEGs. In addition, the PPI network was generated using the STRING database (STRING, V12.0; https://string-db.org) (accessed on 29 December 2023) [87,88] only for DEGs passing the correction for multiple testing. Database search parameters were defined for species set to ‘Homo sapiens’, and the confidence score cutoff was set at 0.4. Other settings remained default. The constructed focused network provided a presentation of the interplay of key DEG products, and their involvement in significantly enriched annotation terms (KEGG, Gene Ontology:Cellular Compartment and Uniprot significant annotated keywords) on the same diagram. Enrichment analysis both for the generated PPI network and annotation terms was performed in the STRING database.

### 4.8. Relative Expression of the Candidate Target Genes by Quantitative Real-Time PCR in a Replication Group of MS Patients

cDNA was prepared using a RevertAid First strand cDNA synthesis kit according to the manufacturer’s protocol (Thermo Fisher Scientific Inc., Waltham, MA, USA) using 1 μg of total RNA. The mRNA levels of the selected target genes were determined by quantitative real-time PCR on an ABI 7500 Fast Real Time PCR System (Applied Biosystems, Inc., Foster City, CA, USA; Thermo Fisher Scientific Inc., Waltham, MA, USA) using TaqMan^®^ gene expression assays: Hs00355782_m1 for *CDKN1A* (Cyclin-Dependent Kinase Inhibitor 1A), Hs00363196_m1 for *EGLN2* (Egl-9 Family Hypoxia-Inducible Factor 2) and Hs01067016_m1 for *MAP1B* (Microtubule-Associated Protein 1B). Relative mRNA levels were normalized by a reference gene, *PPIA* (Peptidylprolyl Isomerase A), Hs99999904_m1. All reactions were performed in duplicates in a 96-optical well plate under the following conditions: 50 °C/2 min (1 cycle); 95 °C/10 min (1 cycle); 95 °C/15 s, 60 °C/1 min (40 cycles).

The relative levels of target genes were calculated using the comparative Ct method [89]. The analysis of relative levels of the candidate genes’ mRNA levels was performed on 2^−dCt^ values. The two unpaired groups of continuous variables that do not follow a normal distribution were compared using the Mann–Whitney *U* test. Values of *p* < 0.05 were considered statistically significant. Statistical analyses were performed using Statistica v8.0 (StatSoft Inc., Tulsa, OK, USA) while graphical presentation of the results was performed using Prism v8 software (GraphPad Software, Inc., Boston, MA, USA).

### 4.9. Quantification of Iron, Transferrin and Ferritin in Serum

After collection of peripheral blood, samples were allowed to clot by leaving blood undisturbed at room temperature for 2 h. The clot was removed by centrifugation at 1000× *g* for 20 min at +4 °C and the supernatant was collected and stored at −20 °C prior to use. Iron concentration in serum samples was determined by spectrophotometry, and transferrin by immunoturbidimetry, using a URIT-8210 Automatic clinical chemistry analyzer (URIT Medical Electronic Co., Ltd., Shenzhen, China). Ferritin serum concentration was determined by immunoturbidimetry, using an AutoLumo A1000 Chemiluminescence Immunoassay System (Autobio Diagnostics Co., Ltd., Zhengzhou, China).

### 4.10. Quantification of GPX4, MDA and 4-HNE in Plasma

Peripheral blood samples, collected with EDTA, were centrifuged at 1000× *g* for 15 min at +4 °C, within 30 min of collection. The supernatant was collected and stored at −20 °C prior to use. A FineTest^®^ Human GPX4 (Phospholipid hydroperoxide glutathione peroxidase) ELISA Kit, Human MDA (Malonyldehyde) ELISA Kit and Human 4-HNE (4-Hydroxynonenal) ELISA Kit (Wuhan Fine Biotech Co., Ltd., Wuhan, China) were used for quantification of GPX4 protein, and MDA and 4-HNE lipid peroxidation products, in plasma samples, respectively. The optical density (OD) of samples was measured at 450 nm by using a HEALES MB-580 microplate reader, and the OD values were used for determination of GPX4 (pg/mL), MDA (ng/mL) and 4-HNE (pg/mL) concentration, from a four-parameter logistic (4PL) curve (https://www.myassays.com/four-parameter-logistic-curve.assay) [90].

## Figures and Tables

**Table 1:** Clinical and biochemical parameters of discovery and replication groups of MS patients with regard to disease course.

Clinical and Biochemical Parameters	Discovery Group			Replication Group		
	RR (N = 24)	SP (N = 24)	*p*	RR (N = 16)	SP (N = 16)	*p*
Age at sampling, y	48.0 ± 6.2	49.8 ± 9.0	0.43	42.8 ± 8.2	48.5 ± 7.6	0.04
Sex, n (m/f)	16/8	7/17	0.004	8/8	8/8	1.00
BMI (kg/m^2^)	23.6 ± 2.4	23.0 ± 3.6	0.51	25.6 ± 5.0	23.9 ± 4.7	0.34
Age at onset, y	31.52 ± 6.26	31.16 ± 9.77	0.89	31.2 ± 8.3	34.3 ± 7.2	0.27
Disease duration, y	16.48 ± 4.64	18.64 ± 7.99	0.33#	11.5 ± 3.4	13.6 ± 7.0	0.40#
EDSS	0.98 ± 0.65	6.60 ± 0.48	0.00#	0.97 ± 0.67	5.56 ± 0.79	0.00#
MSSS	0.47 ± 0.30	7.16 ± 1.10	0.00#	0.94 ± 0.67	6.17 ± 1.56	0.00#
Total number of relapses	1.17 ± 1.34	7.54 ± 3.80	0.00#	2.14 ± 3.41	7.00 ± 4.77	0.00#
Number of relapses in last 2 y	0.13± 0.34	1.28± 1.06	0.0001#	0.12± 0.33	1.31± 0.79	0.00#
GPX4 (pg/mL)	2738.39 ± 1872.95	2862.81 ± 1005.21	0.56#	1970.32 ± 1191.13	1955.91 ± 1387.92	0.78#
MDA (ng/mL)	96.55 ± 25.47	99.91 ± 64.54	0.36#	108.12 ± 47.28	92.88 ± 31.07	0.38#
4-HNE (pg/mL)	1490.10 ± 337.04	1171.19 ± 325.46	0.003	1561.37 ± 279.62	1512.06 ± 278.80	0.58#
Fe (µmol/L)	16.00 ± 3.75	14.60 ± 4.80	0.28	13.65 ± 5.78	14.84 ± 3.39	0.47
Transferrin (g/L)	2.34 ± 0.30	2.35 ± 0.39	0.93	2.61 ± 0.52	2.36 ± 0.25	0.21#
Ferritin (ng/mL)	95.44 ± 100.98	59.3258.98	0.11#	61.29 ± 65.45	85.47 ± 96.49	0.44#

Values are presented as mean ± SD for all parameters, except for Sex; RR—relapsing–remitting multiple sclerosis; SP—secondary progressive multiple sclerosis; MS—multiple sclerosis; GPX4—Glutathione Peroxidase 4; MDA—Malondialdehyde; 4-HNE—4-Hydroxynonenal; Fe—Iron level; *p*—*t*-test; *p*#—Mann–Whitney *U* Test; *p* values < 0.05 were considered statistically significant.

**Table 2:** Ferroptosis-related DEGs in SP compared to RR MS patients.

SP vs. RR DEGs	baseMean	log2FoldChange	lfcSE	*p* Value	*p*adj
*CDKN1A*	9548.4098	0.591158406	0.114681	2.54 × 10^−7^	3.24 × 10^−5^
*EGLN2*	5756.6618	−0.338615318	0.067246	4.77 × 10^−7^	3.24 × 10^−5^
*MAP1B*	75.6888061	0.901324663	0.183941	9.58 × 10^−7^	4.34 × 10^−5^
*SLC7A11*	126.622192	0.606526575	0.132314	4.56 × 10^−6^	0.000155
*SAT1*	19,655.5985	0.4501215	0.099888	6.60 × 10^−6^	0.000179
*SLC11A2*	1181.28541	−0.307764602	0.082231	0.000182	0.004127
*CAT*	4651.63507	0.29174057	0.081601	0.00035	0.006799
*GLUL*	3109.18502	0.380189011	0.11015	0.000557	0.009475
*TP53*	9275.04884	−0.23651102	0.075289	0.001682	0.025412
*GCLC*	3856.85128	0.226106063	0.073082	0.001976	0.02687
*ALOX12*	3420.79701	0.542998884	0.179849	0.002534	0.031335
*NFS1*	1626.194	−0.18163292	0.061148	0.002974	0.033708
*EHMT2*	325.41458	−0.146782661	0.053859	0.006424	0.067202
*CYBB*	22,370.9217	0.299323864	0.113882	0.00858	0.083345
*SEPP1*	55.4441827	0.391031784	0.153583	0.010895	0.087159
*GCH1*	1363.03231	−0.285756188	0.111031	0.010063	0.087159
*BECN1*	7986.9329	0.110282249	0.043312	0.01089	0.087159
*ATF3*	438.943629	0.386548037	0.177873	0.029768	0.202423
*CISD1*	1126.71629	−0.193534746	0.088844	0.029378	0.202423
*PLIN2*	4488.7674	0.216134924	0.101398	0.033044	0.208698
*ALOX5*	7082.81376	0.221153845	0.104174	0.03376	0.208698
*SLC40A1*	13,636.9118	0.250180235	0.119032	0.035571	0.210334
*PSAT1*	455.492259	−0.292751394	0.142061	0.039327	0.222852
*EMP1*	168.560458	0.275828894	0.13874	0.046801	0.2546
*HSPA5 ^#^*	3974.35589	0.198734257	0.063625	0.001787	0.010721
*ATF4 ^#^*	3565.62181	0.199836813	0.063727	0.001714	0.010721

SP vs. RR DEGs—Differentially expressed ferroptosis-related genes in SP compared to RR MS patients; baseMean—average of the normalized count values, dividing by size factors, taken over all samples; log2FoldChange—indicates the gene expression changes in SP compared to RR MS samples on a logarithmic scale to base 2; lfcSE—the standard error estimate for the log2 fold change estimate; *p* value—Wald test *p*-value; *p*adj—*p* value adjusted for multiple testing with the Benjamini–Hochberg procedure which controls false discovery rate (FDR); ^#^—DEGs from the analysis of the highly expressed (“small sub-panel”) ferroptosis-related genes.

**Table 3:** KEGG pathway enrichment analysis of the minimum-order network generated from ferroptosis-related DEGs between SP and RR MS patients.

Pathway	Total	Hits	*p* Value	FDR
Ferroptosis	40	7	2.23 × 10^−9^	5.10 × 10^−7^
Prostate cancer	97	9	3.21 × 10^−9^	5.10 × 10^−7^
Mitophagy—animal	65	7	7.49 × 10^−8^	7.94 × 10^−6^
Hepatitis B	163	9	3.03 × 10^−7^	2.41 × 10^−5^
IL-17 signaling pathway	93	7	9.03 × 10^−7^	5.74 × 10^−5^
Endocrine resistance	98	7	1.29 × 10^−6^	6.84 × 10^−5^
Pathways in cancer	530	13	6.02 × 10^−6^	0.000273
Kaposi’s sarcoma-associated herpesvirus infection	186	8	9.66 × 10^−6^	0.000384
Viral carcinogenesis	201	8	1.70 × 10^−5^	0.000602
Choline metabolism in cancer	99	6	2.05 × 10^−5^	0.000652

Total—number of genes in the pathway; Hits—number of genes in the PPI network involved in the pathway; *p* Value—hypergeometric test; FDR—adjustment for false discovery rate (FDR).

## Data Availability

The datasets supporting the conclusions of this article are included within the article and its Supplementary material. Raw sequencing data will be available upon reasonable request.

## Supplementary Materials

The supporting information can be downloaded at: https://www.mdpi.com/article/10.3390/ijms25053016/s1.
